# Efficacy of TCH/TEC neoadjuvant chemotherapy for the treatment of HER-2-overexpressing breast cancer

**DOI:** 10.3892/ol.2015.2912

**Published:** 2015-01-28

**Authors:** WEICAI CHEN, JINSONG HE, SHUFEN SONG, MIN WANG, HUISHENG WU, XIANMING WANG

**Affiliations:** Center for Breast Disease Diagnosis and Treatment, The Second People’s Hospital of Shenzhen, Shenzhen, Guangdong 518035, P.R. China

**Keywords:** breast cancer, neoadjuvant, chemotherapy, human epidermal growth factor receptor-2 overexpression

## Abstract

The aim of the present study was to observe the efficacy of neoadjuvant trastuzumab combined with docetaxel and carboplatin (TCH), and docetaxel, epirubicin and cyclophosphamide (TEC) chemotherapy in human epidermal growth factor receptor-2 (HER-2)-overexpressing breast cancer. The total cohort of 64 cases of HER-2-overexpressing breast cancer patients was divided into two groups according to their treatment preferences: The TCH group, consisting of 39 patients, and the TEC group, consisting of 25 patients. The neoadjuvant chemotherapy was continued for six cycles prior to comparison of the treatment efficacy. The TCG and TEC groups exhibited an overall response rate of 94.9 and 72.0% (37/39 and 18/25 cases; P<0.05), respectively, and a pathological complete response (pCR; defined as the presence of no invasive or *in situ* residual tumors in the breast) rate of 69.2 and 32.0% (27/39 and 8/25 cases; P<0.05), respectively. Furthermore, no significant differences were identified between the two groups of patients in terms of adverse reactions, such as cardiac dysfunction, bone marrow suppression and liver function impairment. In the present study, the treatment of HER-2-overexpressing breast cancer patients with TCH neoadjuvant chemotherapy demonstrated more favorable efficacy and a higher pCR rate when compared with the TEC-treated group.

## Introduction

Neoadjuvant chemotherapy is an important treatment strategy for breast cancer and a consensus appears to have been reached with regard to its clinical significance. Previous studies have demonstrated that 20–25% of breast cancer patients exhibit human epidermal growth factor receptor-2 (HER-2) overexpression or gene amplification, which results in high biological malignancy and poor prognosis (1,2). HER-2 is a member of the erbB gene family, which is associated with breast cancer (3). HER-2 is expressed during embryonic development and also exhibits an important role in the growth and development of a variety of tissues and organs in adults. HER-2 overexpression in breast cancer most frequently occurs as a result of gene amplification. The overexpression of HER-2 produces a malignant phenotype, which leads to cell proliferation and subsequently a more aggressive tumor phenotype, and thus, patients with HER-2 overexpressing breast cancer exhibit a poor prognosis (4). Furthermore, HER-2 status has been reported to be an independent risk factor for the relapse of the disease (5). Despite marked improvements in the prognosis of HER-2 positive breast cancers, which have been observed due to the widespread use of HER-2-specific therapies, such as trastuzumab, patients continue to exhibit recurrences and disease progression (6). Pathological complete response (pCR) may be used to discriminate between patients with favorable and unfavorable outcomes in response to treatment (7). pCR is a suitable surrogate end point for patients with HER-2-positive tumors (8), and the prognostic impact of pCR is highest in HER2-positive tumors. Trastuzumab-based preoperative neoadjuvant therapy with trastuzumab plus docetaxel and carboplatin (TCH) has previously been reported to achieve a promising efficacy, with a good pCR rate and favorable tolerability in stages II or III in Her-2-positive breast cancer (9). Docetaxel in combination with epirubicin and cyclophosphamide (TAC) has also been used as preoperative systemic therapy for locally advanced breast cancer (10–12). However, the treatment efficiency of TCH and TAC neoadjuvant chemotherapies on Her-2 positive breast cancer has not been comparatively studied. Data from previous studies have demonstrated that among breast cancer driven by HER-2 overexpression, a treatment plan that combines neoadjuvant chemotherapy with trastuzumab may produce a significantly higher pCR rate compared with chemotherapy alone (13,14). In the present study, HER-2-overexpressing patients were administered six cycles of trastuzumab combined with docetaxel and carboplatin (TCH), or docetaxel, epirubicin and cyclophosphamide (TEC) as neo adjuvant chemotherapy prior to surgery. The outcomes were then analyzed in order to compare the clinical efficacy of each treatment.

## Patients and methods

### General clinical data

Between May 2009 and October 2012, the Center for Breast Disease Diagnosis and Treatment (Shenzhen, China) enrolled 64 female patients who had developed breast cancer as a result of HER-2 overexpression into the present study. The patients were all examined by performing a core needle biopsy (CNB) and all diagnoses of breast cancer were established for the first time. The age of the patients varied between 23 and 60 years, with a median age of 38 years. According to the international tumor-node-metastasis (TNM) staging system (15), the cohort included three cases of stage IIB, 21 cases of stage IIIA and 40 cases of stage IIIB breast cancer (Table I). The patients were divided into two groups, according to their treatment preferences, the TCH group (n=39) and the TEC group (n=25). All patients had undergone ultrasonic, X-ray and bone mineral density scans to confirm the absence of distant metastases prior to commencing chemotherapy, and no patients had undergone any treatment, such as chemotherapy, radiotherapy, traditional Chinese medicine or endocrine therapy, prior to admission to the Center for Breast Disease Diagnosis and Treatment. All human studies were approved by the ethics committee of the Second People’s Hospital of Shenzhen (Shenzhen, Guangdong, China), and were thus performed in accordance with the ethical standards of the 1964 Declaration of Helsinki and its later amendments. In addition, all participants provided informed consent prior to their inclusion in the present study.

### Pathological examination and scoring systems

#### Pathological examination

CNB specimens were fixed in 10% neutral formaldehyde solution and paraffin-embedded, then three 4-μm thick sections were cut from each paraffin wax block. The sections were subjected to neuropeptide substance P immunohistochemical analysis (16), with all reagents purchased from Maixin Biotechnology (Fuzhou, China), and routine immunohistochemistry staining was conducted by performing a 2,4-dinitrobenzene chromogenic reaction (17). Furthermore, phosphate-buffered saline was used as the negative control instead of primary monoclonal mouse anti-human HER-2 (dilution 1:1; cat. no. 4B5; Roche, Indianapolis, IN, USA), monoclonal mouse anti-human ER (cat. no. MAB-0062; 1:200; Fuzhou Maixin Biotech Co., Ltd., Fuzhou, China) and monoclonal mouse anti-human PR (cat. no. MAB-0231; 1:200; Fuzhou Maixin Biotech Co., Ltd.) antibodies, and tissue sections positive for estrogen receptor (ER), progesterone receptor (PR) and HER-2 were used as the positive controls.

#### Scoring systems

The ER and PR staining results were classified using a semi-quantitative histochemical scoring system previously established by Allred *et al* (18); however, the present study only considered the intensity of the stained cells. The intensity score ranged from 0 to 3 as follows: 0, no staining (−); 1, weak staining (+); 2, intermediate staining (++); and 3, intense staining (+++), with positive expression noted as ER(+) or PR(+) and a lack of positive staining noted as ER(−) or PR(−). Furthermore, Her-2/neu protein expression levels were scored on a scale of 0 to 3, according to a system previously utilized by Almasri and Al Hamad (19). The cellular staining was scored as follows: (i) Negative (−), no membrane staining or staining in <10% of the tumor cells; (ii) weak positive (+), weak focal membrane staining in >10% of the tumor cells; (iii) intermediate (++), weak to moderate complete membrane staining in >10% of the tumor cells; and (iv) strongly positive (+++), weak to moderate cytoplasmic reactivity and intense membrane staining in >10% of the tumor cells. In addition, HER-2(−) or HER-2(+) represented a lack of HER-2 overexpression and HER-2(+++) indicated HER-2 overexpression. HER-2(++) results were double-checked by performing fluorescence *in situ* hybridization (FISH), using FISH(+) as HER-2 overexpression and FISH(−) as no HER-2 overexpression (20).

### Treatment strategy

#### TCH regimen

On day one (the first day of each chemotherapy cycle), the TCH regimen was administered as 75 mg/m^2^ docetaxel intravenously, with a carboplatin area under the curve of six. Trastuzumab was intravenously infused once a week at 4 mg/kg for the first week, 2 mg/kg for the following 16 weeks (17 weeks total) and 6 mg/kg every three weeks for one year after surgery. Cardiac function was monitored at baseline, and at three, six and nine months after day one.

#### TEC regimen

Docetaxel was administered via an intravenous drip at a dose of 75 mg/m^2^ on day one. On day two, 80 mg/m^2^ epirubicin was intravenously administered along with 500 mg/m^2^ cyclophosphamide.

For the two regimens, one chemotherapy cycle lasted for 21 days and six cycles were conducted in total. Two weeks after the completion of the neoadjuvant chemotherapy regimes, modified radical mastectomy or breast conserving therapy were performed.

### Evaluation of treatment efficacy

For the patients in the two treatment groups, magnetic resonance imaging was employed prior to and following the treatment to determine the size of the tumor and to evaluate the efficacy of each chemotherapy regime.

According to the uniform criteria established by the World Health Organization, the efficacy of the treatment strategies was classified as follows: Clinical complete response (cCR), disappearance of all tumors/lesions upon clinical examination; partial response (PR), ≥50% decrease in tumor size; stable disease (SD), <50% decrease in tumor size or <25% increase in tumor size; and progressive disease (PD), ≥25% increase in tumor size or development of new lesions (21). Additionally, overall response (OR) was calculated as CR plus PR, and pCR was defined as the presence of no invasive cancerous cells at the *in situ* tumor region of the surgical specimen, with no concurrent metastasis in the axillary lymph nodes.

### Statistical analysis

SPSS software (version 17.0; SPSS Inc., Chicago, IL, USA) was used to perform the χ^2^ test on the data and P<0.05 was considered to indicate a statistically significant difference.

## Results

### Groups

Her-2/neu was shown to be overexpressed in all patients [+++: 45; ++: 14; +: 5, 19 cases (Her-2++/+) were double-checked by FISH and were demonstrated to be Her-2/neu overexpression FISH(+)]. The cohort of 64 patients was randomly separated into two groups: The TCH group, consisting of 39 patients, and the TEC group, consisting of 25 patients. No significant differences in age, tumor staging or pathological characteristics were identified between the two groups.

### Curative efficacy

The TCH and TEC groups exhibited OR rates of 94.9 and 72.0%, respectively (P<0.05), and pCR rates of 69.2 and 32.0%, respectively (P<0.05) (Table II). These results indicated that TCH chemotherapy was more efficacious than TEC chemotherapy for the treatment of HER-2 overexpressing breast cancer.

### Adverse reactions

No treatment-associated mortality or cardiac insufficiency occurred in any of the patients enrolled in the present study. Eight patients in the TEC group exhibited symptoms, including shivering and fever, upon the initial infusion of trastuzumab; however, these adverse effects were remitted once the remainder of the treatment was applied.

All patients experienced myelosuppression to varying degrees, and the ratio of grade III-IV neutropenia in the TCH and TEC groups was 25.6% (10/39 cases) and 28.0% (7/25 cases), respectively, demonstrating no statistical significance (P=0.928). Treatment proceeded when the leukocyte level was recovered following granulocyte-colony stimulating factor (G-CSF) therapy.

The patients in the two groups demonstrated different levels of gastrointestinal reactions, such as nausea, vomiting and diarrhea, which were relieved following antiemetic treatment and fluid supplementation. In addition, patients in the two groups only exhibited oral ulcers, rash, peripheral neurotoxicity and hepatic dysfunction to a mild extent, which were tolerable after expectant treatments.

## Discussion

Neoadjuvant chemotherapy, with its broad and ever-increasing number of applications, is now substantially important in integrated therapies for breast cancer. Previous studies have revealed that neoadjuvant chemotherapy, compared with traditional adjuvant chemotherapy, may eliminate early subclinical disseminated lesions, and reduce the TNM stage of primary lesions and regional lymph nodes (22,23). Neoadjuvant chemotherapy may additionally provide information on the chemotherapeutic sensitivity of the tumor and the prognosis of patients, establishing it as particularly effective in the treatment of local terminal breast cancer. Neoadjuvant chemotherapy has become one of the standard therapeutic regimens for the treatment of late clinical stage breast cancer (24) and was therefore used in the present study, in which 95.3% (61/64 cases) of patients were in the late clinical stages, including 21 stage IIIA patients and 40 stage IIIB patients.

Previous clinical studies have indicated that breast cancer patients with HER-2 overexpression typically demonstrate a poor prognosis and a high degree of malignancy in tumorous biological behavior (25,26). In recent years, the increasing clinical application of trastuzumab has significantly improved the disease-free and overall survival (OS) rate of patients (27). Although trastuzumab is predominantly employed post-operatively or in the treatment of metastatic HER-2-overexpressing breast cancer, clinicians worldwide have hypothesized that a trastuzumab-associated chemotherapeutic scheme may enhance the pCR of HER-2-overexpressing breast cancer patients by a large margin (28,29).

In the BCIRG006 clinical trial (29), a TCH regimen of trastuzumab combined with docetaxel and carboplatin was designed. The three-year median follow-up period indicated that the efficacy of this joint chemotherapy regimen of trastuzumab and non-anthracyclines was similar to the regimen of doxorubicin and cyclophosphamide with sequential docetaxel and trastuzumab (AC-TH), but with fewer cardiac toxicity reactions. Additional studies indicated that trastuzumab and docetaxel exhibit a synergistic effect when administered together, markedly improving the response rate of HER-2-overexpressing breast cancer by increasing the time to progression, as well as the median survival time (30–33). Furthermore, another study reported that the efficacy of neoadjuvant chemotherapy combined with trastuzumab was significantly greater than that of chemotherapy alone in the treatment of HER-2-overexpressing patients (pCR, 31.7 vs. 15.7%) (34). Currently, pCR is the most important indicator for assessing the efficacy of neoadjuvant chemotherapy regimens (35,36). The results of the present study indicated that the efficacy of treating 39 HER-2-overexpressing breast cancer patients with the TEC regimen was most favorable, demonstrating 94.9% OS and 69.2% pCR rates after six cycles of observation. This high pCR rate may be explained by the previous medical history of enrolled patients, the timely and effective treatment of side-effects and completion of the six-cycle treatment by all patients.

Major adverse reactions are caused by the use of trastuzumab and chemotherapeutic agents. Of the reactions caused by trastuzumab, fever appears to be the most common response, predominantly occurring during the first infusion as opposed to after the administration of medication. Remission of fever may be achieved via the application of conventional antipyretic analgesics and anti-allergy medicines. However, cardiac dysfunction is the most severe type of adverse reaction, and may manifest as dyspnea, pulmonary edema, peripheral edema and cardiac dilatation (37). In a previous study, the rate of symptomatic heart failure was 4–6% when trastuzumab was used alone, however, the rate was significantly higher (27%) when combined with anthracyclines (38).

Adverse reactions caused by the administration of chemotherapeutic agents include myelosuppression to various degrees, gastrointestinal reactions, oral ulcers and alopecia (39); however, in the present study, none of the 39 patients in the TCH group experienced severe toxic side-effects or cardiac insufficiency. The underlying explanation for this may be that the enrolled subjects had no history of heart disease, the observation of a ≥50% left ventricular ejection fraction upon ultrasonography or the application of an anthracycline-free neoadjuvant chemotherapy scheme. Despite the lack of severe side-effects, patients in the two groups did experience grade III-IV neutropenia, with treatment commencing following G-CSF treatment and a recovery of leukocyte levels. Other adverse reactions were comparatively mild, and all were tolerated following the administration of expectant and supporting treatments.

In conclusion, the neoadjuvant chemotherapy regimen of trastuzumab combined with docetaxel and carboplatin exhibited significant efficacy, tolerable adverse reactions and a high pCR rate in the HER-2-overexpressing breast cancer patients. Thus, the TCH regimen should be applied, particularly for the treatment of late clinical stage breast carcinoma.

## Figures and Tables

**Table I:** General clinical data of all breast cancer patients (n=64).

	Cases	
	
Parameter	n	%
TNM stage		
IIB	3	4.7
IIIA	21	32.8
IIIB	40	62.5
Pathological type		
Invasive ductal carcinoma	44	68.8
Infiltrating lobular carcinoma	20	31.2
Receptor expression		
ER(+)/PR(+)	23	35.9
ER(+)/PR(−)	13	20.3
ER(−)/PR(+)	12	18.8
ER(−)/PR(−)	16	25.0
Axillary lymph node metastasis		
Negative	7	10.9
Positive	57	89.1

TNM, tumor-node-metastasis; ER, estrogen receptor; PR, progesterone receptor.

**Table II:** Comparison of OR and pCR rates of the two treatment groups.

	Regimen, % (n)			
			
Efficacy	TCH (n=39)	TEC (n=25)	χ^2^	P-value
OR	94.9 (37)	72.0 (18)	4.838	0.028
pCR	69.2 (27)	32.0 (8)	7.085	0.008

OR, overall response; pCR, pathological complete response; TCH, docetaxel, carboplatin and trastuzumab; TEC, docetaxel, epirubicin and cyclophosphamide.
